# Myocardial Opioid Receptors in Conditioning and Cytoprotection

**DOI:** 10.3390/ph4030470

**Published:** 2011-03-04

**Authors:** Grant Williams-Pritchard, John P. Headrick, Jason N. Peart

**Affiliations:** Heart Foundation Research Centre, Griffith University, PMB 50 Gold Coast Mail Centre, QLD 9726, Australia

**Keywords:** opioids, preconditioning, postconditioning, cross-talk

## Abstract

Opioid compounds and G-protein coupled opioid receptors (ORs) have been studied widely in terms of central nervous system (CNS) actions relating to pain management and drug abuse. Opioids are also linked to induction of mammalian hibernation, a natural state of tolerance involving prolonged and orchestrated shifts in cellular metabolism, growth and stress resistance. It is not surprising then that OR agonism induces acute or delayed cytoprotective states in myocardium, rendering ORs an attractive target for protection of cardiac tissue from the potentially fatal consequences of ischemic heart disease. Cardiac ORs are implicated in triggering/mediating so-called ‘conditioning’ responses, in which powerful cytoprotection arises following transient receptor ligation prior to or immediately following ischemic insult. These responses involve one or more OR sub-types engaging pro-survival kinase cascades to ultimately modulate cell stress and mitochondrial end-effectors. However, important questions remain regarding the role of endogenous opioids, OR signalling, and the transduction and mediation of these protective responses. We briefly review opioid-mediated cardioprotection, focussing on recent developments in signal transduction, the role of receptor ‘cross-talk’, and the effects of sustained OR ligand activation.

## 1. Introduction

Opioids modulate cellular function via G_i/o_ coupled members of the G-protein protein coupled receptor (GPCR) superfamily—the μ-, κ-, and δ-ORs. They are activated by opioid peptides derived from the endorphin, dynorphin and enkephalin families, share ∼50% homology, and differ in binding properties, tissue distribution, and cell signalling. The μ-ORs appear sensitive to endorphins, κ-ORs preferentially bind dynorphins, while δ-ORs have a higher affinity for enkephalins. Pharmacological evidence suggests that the κ-, and δ-OR sub-families may include κ_1_, κ_2_, δ1, and δ_2_ sub-types. For a comprehensive review of OR pharmacology specifically within the cardiovascular system, readers are directed to comprehensive review [1].

While initially considered to act indirectly via modulation of nervous/sympathetic control, it is known that myocardial cells are sites of opioid peptide synthesis, storage and release [2]. Stressors such as ischemia elevate myocardial peptide turnover [3], and κ- and δ-ORs are expressed in myocardium, whereas binding and gene expression studies excluded μ-ORs from adult myocardium [4,5]. However, immunofluorescence microscopy showed co-localization of μ-opioid receptors with Cav-3 in both sarcolemmal and intracellular membranes of the adult myocyte [6]. Ventricular myocardium actually contains the highest levels of preproenkephalin (enkephalin precursor) mRNA in the body, surpassing that for the central nervous system [7]. Myocardial expression of opioids and ORs is consistent with opioidergic regulation of cardiovascular function and myocardial stress resistance. This review focuses specifically on the roles of OR in myocardial cytoprotection.

## 2. Opioid Receptor-Mediated Cardioprotection

Endogenous opioids possess autocrine/paracrine functions within the heart and vessels. For example, OR activity inhibits excitation-contraction coupling, modulates vascular tone, may play a role in cardiogenesis, and exerts potent cytoprotective actions in the heart. Recent work indicates endogenous opioids play a role in protecting cardiac tissues from ischemia-reperfusion (I/R) injury [8], and in mediating ischaemic preconditioning [9-11]. Other studies suggest opioids function as ‘mediators’ rather than ‘triggers’ of acute preconditioning [12].

## 3. Involvement in Pre- and Post-Conditioning

Two of the most intensely studied protective modalities are the conditioning responses—pre- and postconditioning. Preconditioning was discovered by Murry *et al.* [13], and refers to induction of both acute and delayed protective states in response to a transient episode of ischemia prior to prolonged insult. The transient ischemia can be replaced by transient agonism of GPCRs implicated in this response [14]. Protection against infarction with postconditioning was established by Vinten-Johannsen and colleagues, who documented protective actions of brief episodic ischemia during the first minutes of reperfusion following sustained insult [15], extending earlier observations of electrophysiological protection with intermittent reperfusion [16]. These responses have garnered considerable interest as potentially clinically relevant protective stimuli [17], underpinning extensive interrogation of underlying mechanisms. Despite some conflicting findings, these studies identify roles for opioids and ORs in induction or mediation of conditioning responses.

Pre-ischemic OR agonism mimics ischemic preconditioning [18], antagonists of ORs counter the protection with preconditioning when applied prior to the ischemic preconditioning stimulus, in an acute setting [19] or during the index ischemia in a delayed preconditioning model [20]. Thus, there is some support not only for a role for ORs in the initial trigger phase of preconditioning, but also in subsequent mediation of protection during subsequent ischemia-reperfusion.

Consistent with mechanistic links between preconditioning and more recently studied postconditioning, evidence also supports an essential role for ORs in postconditioning. Beneficial effects of ischemic post-conditioning are replicated by OR activation, and countered by δ-OR antagonism [21]. Furthermore, Zatta *et al.* [22] presented evidence implicating both μ- and δ-ORs in cardioprotection afforded by ischemic postconditioning, and showed protection was associated with preservation of myocardial enkephalin levels (particularly the precursor proenkephalin). In contrast, a recent study in a similar model reports that κ- and δ-ORs but not μ-ORs mediated ischemic postconditioning [23]. Reasons underlying these differences are unclear, though may potentially involve dose-dependent selectivity of pharmacological tools employed. Analysis of protection of the brain via remote postcondtioning (triggered in response to ischemia in remote limbs or organs) also supports protection via intrinsic OR activity [24], though this is yet to be established for remote cardiac postconditioning.

As with opioidergic preconditioning, exogenous activation of κ- and δ-ORs at reperfusion affords protective postconditioning [25-28], and underlying mechanisms mirroring those for ischemic conditioning responses. Studies thus support recruitment of the archetypal PI3k and GSK3β signalling axis [26,27,29], phosphorylation of eNOS and NO production [28], regulation of mitochondrial and sarcolemmal K_ATP_ channel opening [26,27,29], and inhibition of mPTP function, perhaps through a NO-cGMP-PKG path [21]. However, multiple pathways to cardiac protection have been identified, including the Reperfusion Injury Salvage Kinase (RISK) [30] and Survivor Activating Factor Enhancement (SAFE) [31] paths. In this respect, there is also evidence for JAK-STAT involvement and modulation of BCL-2 expression and apoptosis [32], as in the SAFE signalling model. Whether these different signal paths are distinct or do indeed interact and/or converge on end-effectors is at present unclear.

### 3.1. Downstream Effectors of Opioid Mediated Cardioprotection

As detailed previously [33,34], conventional models link acute OR activation to protein kinase cascades, reactive oxygen species (ROS) generation, and modulation of mito K_ATP_ channel controlling mPTP opening [35-39]. Whether the latter channels are ‘end-effectors’ or proximal to end-effectors is still debated, as is the contribution of sarcolemmal channels [36,40,41].

ORs couple to G_i/o_ proteins to inhibit adenylyl cyclase, with δ- and κ-ORs known to activate PLC [42] and phosphoinositol turnover [43]. Additionally, OR agonism activates tyrosine kinase and PKC, perhaps in parallel [36,44], and leads to opening of both sarcolemmal and mito K_ATP_ channels [37,38]. ORs also regulate ion channels via G-protein interactions [45,46]. In terms of cardioprotection, infarct limitation with δ-OR agonism is PKC- and NOS-dependent [44,47], and involves tyrosine kinase (TK) and MAPK signalling [36,44,48]. Acute OR protection during reperfusion is dependent upon PI3-K, target of rapamycin (mTOR), and GSK3β modulation [49]. Collectively, data implicate non-Src-dependent TK, extracellular signal-regulated kinase (ERK1/2) and PI3K/PKC pathways as integral signalling components of acute δ-*OR* mediated cardioprotection.

**Figure 1:**
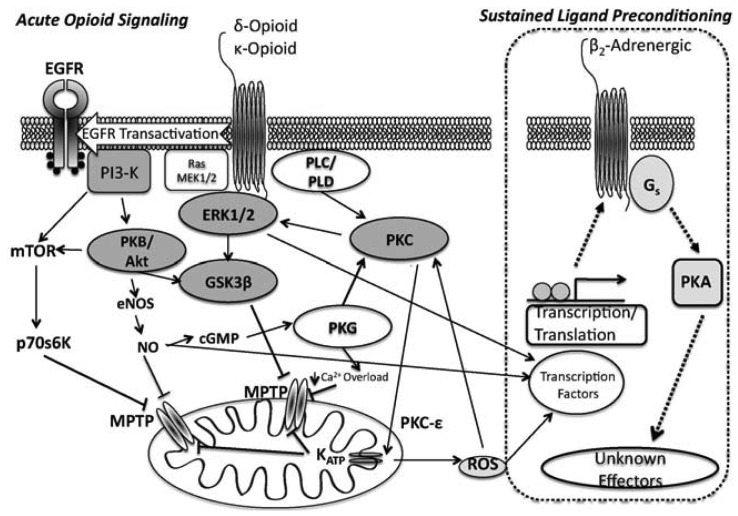
Generalized signaling scheme for acute- and sustained ligand opioid-dependent cardioprotection. Sustained ligand preconditioning with opioids appears to share common ‘acute’ signaling during the induction phase of the phenotype. Shaded text identifies proteins shown to be involved in opioid-mediated cardioprotection. Dashed arrows imply unknown pathways/mechanisms of activation in sustained ligand activated preconditioning.

Signalling in acute κ-OR dependent cardioprotection is less well defined: κ-OR inhibition of ventricular cardiomyocyte shortening appears to be G_i_-dependent [50], and κ-ORs suppress cardiomyocyte cAMP accumulation via a phosphoinositol/Ca^2+^ path [51]. Downstream effectors affording cardioprotection from κ-OR activation likely mimics that of the δ-OR, (PI3k/Akt, PKC and mito K_ATP_ channel dependent, [26,52]) (Figure 1).

There is little information regarding any potential role for the μ-ORs in the setting of cardioprotection. Until recently it was thought that the μ-OR was absent from the mature myocardium [4-6]. Indeed, there is a lack of evidence supporting a role for the μ-OR in preconditioning. However, Zatta *et al.* [22] recently reported that ischemic postconditioning was mediated through endogenous μ-OR activation. The involvement of μ-OR in conditioning the heart is controversial at best. As such, detail regarding potential mechanisms is absent.

### 3.2. Caveolae and ORs

Immunohistochemical analysis co-locates μ-ORs and caveolin-3 (Cav-3) in sarcolemmal and intracellular membranes of the adult myocyte, and caveolins appear essential to OR cytoprotection [6]. Caveolae, microenvironments enriched in caveolin, cholesterol and sphingolipids, form characteristic flask-like invaginations of the membrane. Caveolar structural proteins (caveolins-1, -2 and -3) may act as scaffolding molecules to localise and regulate interactions between receptors, signalling components, and effectors, facilitating coordinated regulation of cell function [53]. This not only impact on sarcolemmal targeting, but transduction of protective signals to mitochondria [54]. Importantly, caveolae are critical to I/R tolerance and cardioprotection: protective stimuli increase caveolae formation, cardioprotection is blocked by caveolar disruption, caveolin-3 (cav-3) knockout (KO) eliminates preconditioning [55,56], and overexpression of cav-3 boosts intrinsic I/R tolerance [56].

In adult cardiac myocytes, ORs are localised to cav-3-associated domains [6], and caveolar disruption abolishes the protective effects of δ-OR activation [57]. Interestingly, an *in vivo* model utilising cav-3 overexpression and knock-out mice confirmed cav-3 dependence of δ-OR protection, and revealed that protection conferred by cav-3 overexpression was negated by naloxone [58]. This supports an integral relationship between caveolae, caveolins and ORs, with δ-ORs specifically implicated in the cytoprotective capacity of caveolae. Caveolar localisation of ORs may also contribute to potential cross-talk between ORs and other protective GPCRs.

## 4. Opioid Receptor Cross-Talk and Cardioprotection

The OR family engages in cross-talk between its own members and with other receptors. Action of ORs may involve modulation of or dependence upon the function of other receptor types and vice versa. Cross-talk may be indirect through trans-regulation of downstream signalling and the effects of heterologous sensitisation and desensitisation, or more direct, in the context of receptor-receptor interactions and GPCR dimerisation. The ORs are known to form homo- and heterodimers in heterologous cell lines and non-cardiac tissue: δ-ORs can heterodimerise with both κ- and μ-ORs, altering pharmacological properties, and heterodimers may also form between ORs and other GPCRs, including somatostatin, substance P, and α_1_ and ß_2_-adrenergic receptors. Specific studies in cardiovascular tissue are lacking, though such complexes could impact on cardiac control and underpin forms of OR cross-talk.

One of the earlier reported forms of myocardial OR cross talk involves δ-OR modulation of ß-adrenergic responsiveness, limiting norepinephrine-induced increases in sarcolemmal L-type Ca^2+^ current, cytosolic Ca^2+^ transients, and contraction in isolated ventricular myocytes [59]. These results were confirmed by Pepe *et al.* [60] in intact hearts, demonstrating cross-talk between δ-OR and ß_1_-adrenoceptor signaling, with ORs inhibiting adenylyl cyclase via a PTX-sensitive G_i/o_ protein. The cAMP-independent inotropic effects of ß_2_-AR agonism appear insensitive to δ-OR activity. In terms of cardioprotection, δ-OR mediated infarct reduction is negated by ß_2_-adrenoceptor blockade [61]. These investigators also reported an essential requirement for intrinsic cardiac adrenergic cells in δ-OR protection of cardiac myocytes, supporting reliance on endogenous epinephrine/ß_2_-adrenoceptor signaling. A subsequent study [62] added calcitonin gene-related peptide receptors (CGRP-R) to the interaction between δ-OR and ß-adrenoceptors, reporting synergistic cardioprotection through a δ-OR regulated ß_2_-adrenoceptorAR/CGRP-R co-signaling in cardiac adrenergic cells. Our own data indicates that mediation of protection with sustained opioid agonism (see below) is ß_2_-adrenoceptor dependent [63]. ß-adrenoceptor and OR cross-talk appears to involve modulation of G-protein signaling and kinase activity, but could also involve dimerisation: both δ- and κ-ORs physically associate with ß_2_-adrenoceptors when co-expressed in HEK-293 cells [64]. As a result, δ-OR activation mediates ß_2_-adrenoceptor internalisation and inhibits ß_2_-adrenoceptor triggered ERK1/2 activation.

Our prior work highlights cardioprotective cross-talk between OR and adenosine receptors. In an initial study [65], the infarct sparing effects of OR stimulation in rats were abolished by an adenosine A1 receptor (A_1_AR) antagonist, and the reverse was also found to be true (OR blockade attenuated cardioprotection in response to A_1_AR activation). In a subsequent study, the protective effects of increased endogenous adenosine (following adenosine kinase inhibition) were found to be dependent upon δ-OR activity [66]. The basis of this cross-talk, which evidences an essential role for both receptors in responses to either, remains to be established. There is evidence of positive cross-talk between AR and ORs in other models, which may reflect modulation of endogenous ligand levels and distal signalling: Kaster *et al.* [67] report that anti-depressant actions of A1 and A_2_ARs involve κ- and μ-OR activities; and remote conditioning effects of intrathecal ORs require both central and peripheral AR activities [68]. While these effects may involve modulation of endogenous ligand signaling, AR/OR receptor complexes could contribute. There is evidence that ARs are essential for synergistic signaling between δ-OR, μ-ORs, CB1 and D_2_ receptors in nervous tissue (with A1 or A_2A_ activity and Gi-βγ required for this synergistic action) [69,70]. The mechanism underlying AR-dependent hypersensitisation of co-expressed OR receptors is not known, but appears to centre on priming of adenylate cyclase and increased signaling activation. However, while studies in heterologous cell lines support receptor-receptor interactions between A_1_ARs and δ-ORs, their nature is distinct from responses observed in native cardiac tissue: in a CHO cell model, co-expressed A_1_ARs heterologously desensitize δ-OR mediated kinase signaling, and induce phosphorylation of δ-ORs [71]. Our data, in contrast, supports an essential requirement for both AR and OR activities in cardioprotection. In recent studies in murine HL-1 cells [72], we also find that intracellular kinase signaling (ERK1/2) and AR and OR mRNA expression are similarly co-regulated via A_1_ARs and δ-ORs, with antagonism of either receptor alone negating responses to both. Providing a link between the two receptors, signal activation by either A_1_AR or δ-ORs appears commonly dependent upon EGFR function. Indeed, δ-OR post-conditioning of the myocardium has been shown to depend upon transactivation of the EGFR [73]. Thus, ORs and ARs may both engage a common EGFR-dependent pathway to activate downstream protective signals, which may contribute to observed cross-talk.

In addition to these forms of cross-talk, one can consider δ-ORs as signaling intermediates involved in transducing cardioprotection. It was recently demonstrated that epicatechin, an antioxidant flavonoid with no known direct receptor-mediated activity, produced profound δ-OR dependent protection against infarct development [74]. Protective effects of epicatechin were associated with increased phosphorylation of pro-survival kinases (Src, Akt, IκBα) and decreased pro-apoptotic protein expression, both effects countered by δ-OR antagonism. Similarly, infarct sparing actions of the epoxyeicosatrienoic acid 11,12-EET, for which a specific EET membrane receptor is yet to be identified, are reversed by either δ- or κ-OR inhibition [75]. Irrespective of the specific molecular basis of these cross-talk effects, such reports further highlight the importance of intrinsic OR activity in transduction of cardioprotection.

## 5. Sustained Opioidergic Preconditioning

In 2004, we described a protective phenomenon dubbed chronic morphine preconditioning [76], which we more accurately label as sustained ligand-activated protection (SLP), since we show the response involves selective δ-opioid receptor (δ-*OR*) activation [77]. SLP can be induced by 3–5 days of δ-*OR* activation, mediating protection that exceeds that with preconditioning or postconditioning and persists for at 5 days (perhaps ≥7 days) [77]. Signalling is unique *vs.* that for preconditioning or postconditioning [63].

From a clinical standpoint, generation of sustained protected states (as in SLP) has advantages [78]. Precise timing of treatment relative to I/R becomes less critical, and the need for ongoing therapy is reduced. Prolonged protection might be particularly useful in prophylactic therapy in high-risk patients, and for limiting time-dependent progression of injury during or after surgery (for example). Temporal properties of classic preconditioning are sub-optimal: despite rapid induction, the powerful initial window is brief (1–2 h), while the later sustained window is less efficacious and lasts only 2–3 days. With postconditioning, protection arises rapidly yet does not impact on ischaemic injury, the window of opportunity for protection is likely narrow, and it is unclear if protection is long-lasting (or effective in aged hearts).

Downey's group initially addressed the possibility of sustained cardioprotection (via continuous A_1_ adenosine receptor agonism) [79], and Dana *et al.* [80] subsequently showed it was possible to generate persistent protection for 10 days with repetitive A_1_ receptor agonism. Inagaki *et al.* [81] described sustained protection following 10-day infusion of a PKC-ε activator. We document protection for at least 5 days following acute irreversible δ*-OR* agonism [82]. Thus, prolonged protected states can be generated in cardiac tissue, though are yet to be exploited clinically to limit injury.

The SLP response [76,77,83] affords protection exceeding that for conventional preconditioning, and persists for up to 7 days after removal of the initiating stimulus [77]. We showed that SLP depends upon signalling distinct from so-called RISK paths [30,63] that mediate preconditioning and postconditioning, likely explaining retention of SLP efficacy with aging [34] *vs.* failure of the latter responses [84-88]. Inhibition of downstream kinases (including PI3-K/Akt, PKC) and mito K_ATP_ channels *all* fail to block mediation of SLP during acute I/R [63]. Rather, mediation of SLAP is G_s_- *vs.* G_i_-dependent, requires protein kinase A (PKA), and depends upon ß_2_-adrenoceptor activity [63] (Figure 1). These findings are consistent with emerging roles for PKA in cardioprotection [89-91]. ß_2_-adrenoceptor involvement is interesting, since ß_2_-adrenoceptors favour cell survival, limiting I/R injury and apoptosis in a G_i_/PI3-K (*vs.* G_s_) dependent manner [92,93]. Inhibitory effects of ß_2_-adrenoceptor antagonism against SLP protection may reflect shifts in effector coupling or PKA activation in SLP hearts.

Certainly this is documented for the ß_2_ receptor, though not strictly in accordance with these observations. For example, receptor phosphorylation by PKA alters G-protein selectivity of ß_2_-adrenergic receptors, favoring coupling to G_i_ *vs.* G_s_ protein, and reversing the effects of the receptor on cAMP generation [94]. Phosphorylation-dependent switching of G-protein coupling allows the receptor to engage alternate signaling (e.g., G_i_-dependent MAPK activation). Cardiomyocyte ß_2_ (but not ß_1_) receptors favor cell survival via pertussis toxin sensitive G_i_ signaling, PI3K and Akt.

In contrast to the mediation phase (*i.e.*, the period of ischemia-reperfusion when protection is expressed), the induction of the SLP phenotype is δ-opioid receptor mediated and is induced in a PI3K-dependent/PKA-independent manner [77]. This is interesting, as it supports distinct phases to the response, with PI3K/Akt involvement during induction vs. mediation, and PKA involvement during mediation. It seems that sustained OR agonism induces changes in signaling that may switch OR responses from Gi to Gs dependent mechanisms, and involves PI3K- and PKA signalling (albeit, temporally distinct). These findings are also somewhat consistent with the role for OR signalling in hibernation.

## 6. Opioids and Hibernation

Opioids, and in particular the δ-receptor sub-type, are strongly implicated in mammalian hibernation. Hibernation can be induced or reversed by δ-opioid agonists and antagonists, respectively, even in species that do not normally hibernate. Hibernation is thought to be triggered by changes in serum levels of a δ-opioid like peptide, termed ‘hibernation induction trigger’ (HIT) [95]. HIT and the δ-opioid peptide DADLE can induce hibernation like states in non-hibernating mammals, and mammalian hibernation is associated with an improvement in tissue resistance to stressors such as hypoxia.

Many parallels exist between SLP and hibernation: hibernation is normally triggered by endogenous δ-opioid agonism, which can also induce hibernation in non-hibernating primates [96,97]; δ-opioid-triggered hibernation increases cellular stress resistance [98,99]; hibernating and anoxia-tolerant species specifically harness PKA-dependent signaling [100,101]; and repression of Akt may suppress energy-costly anabolic/growth processes to maintain cell viability over extended hibernation periods [102,103]. The protected SLP phenotype is induced by prolonged δ-opioid agonism, involves PI3K dependent signals with early and late repression of Akt expression (both total and phosphorylated) [77], leading to sustained PKA-dependent cardioprotection. We also unexpectedly found that prolonged PI3K/Akt inhibition with wortmannin induces some ischemic tolerance. Together, these speculative data hint at cardioprotection in response to sustained PI3K/Akt repression, consistent with the role for Akt in δ-opioid-mediated cytoprotective hibernation.

## 7. Summary

The opioid system of peptides and receptors have been shown to evoke profound cytoprotective states, from intrinsic/endogenous examples such as hibernation, through to exogenous pharmacological manipulation of receptors such as a post-conditioning mimetic. While the mechanisms may not be fully understood, they appear to mirror those of ischemic preconditioning (involving a signaling axis incorporating PI3k, GSK3β, K_ATP_ channels and the mPTP). Acute opioid-mediated protection also appears dependent upon activated adenosine receptors. Moreover, opioids can confer an extended window of cardioprotection. As opioids are currently used both post-operatively and for both acute and chronic pain, a long period of drug development before opioids will be approved for use as cardioprotective agents would not be required.
